# Ecological Dynamics and Microbial Treatments against Oomycete Plant Pathogens

**DOI:** 10.3390/plants10122697

**Published:** 2021-12-08

**Authors:** Karen E. Sullam, Tomke Musa

**Affiliations:** Ecological Plant Protection in Arable Crops, Agroscope, 191 Reckenholzstrasse, 8046 Zurich, Switzerland; tomke.musa@agroscope.admin.ch

**Keywords:** microbial biocontrol agents, oomycete, plant pathogens, community assembly, spatial dynamics

## Abstract

In this review, we explore how ecological concepts may help assist with applying microbial biocontrol agents to oomycete pathogens. Oomycetes cause a variety of agricultural diseases, including potato late blight, apple replant diseases, and downy mildew of grapevine, which also can lead to significant economic damage in their respective crops. The use of microbial biocontrol agents is increasingly gaining interest due to pressure from governments and society to reduce chemical plant protection products. The success of a biocontrol agent is dependent on many ecological processes, including the establishment on the host, persistence in the environment, and expression of traits that may be dependent on the microbiome. This review examines recent literature and trends in research that incorporate ecological aspects, especially microbiome, host, and environmental interactions, into biological control development and applications. We explore ecological factors that may influence microbial biocontrol agents’ efficacy and discuss key research avenues forward.

## 1. Introduction

Host-associated microbiota are integral parts of organismal biology and have a strong impact on host physiology [1,2]. The microbiome, which includes the microbiota, their collective genetic material, as well as their collective functions and properties [3,4], represents a novel resource for disease prevention and treatment in plant and animal health [5,6,7]. It is increasingly examined in a context of ecological and evolutionary concepts [8,9]. The inclusion of a comprehensive microbiome perspective is revolutionizing a number of fields, including medicine, agriculture, forestry, and evolutionary biology [10,11,12,13,14]. Within human medicine, recent advancements in microbiome research are changing the understanding of certain diseases and methods to treat them [15,16]. In order to establish and maintain a healthy microbiome, human disease treatments now consider ecological principles regarding microbial colonization and community establishment to treat diseases without antibiotics [17,18,19]. New efforts are driving the use of naturally occurring microbiota to manipulate the microbiome for the maintenance of weight, mental health, and reduction of certain diseases [20,21,22,23]. Similar to the development of perspectives within human health fields, microbiota-based treatments are increasingly considered as viable sources for disease control in plants and may help to decrease synthetic inputs [6,11,24]. This review explores challenges associated with oomycete diseases in plants, their relationship to the microbiome, and possible avenues of using the microbiome to help suppress diseases.

The oomycetes are a mostly pathogenic class, of which 60% of known species are plant pathogens [25], but members also parasitize fish, crustaceans, insects, humans, and other vertebrates [26,27,28]. The lineage has independently evolved pathogenicity in plants and other organisms several times, and it includes members with diverse lifestyles ranging from asymptomatic endophytes to obligate biotrophic pathogens [25,29]. Members of the oomycetes cause some of the most problematic plant diseases, including *Phytophthora infestans* (potato late blight), *Plasmopara viticola* (grapevine downy mildew), and *Pythium* spp. (cause of damping off and root rot), and can severely reduce agricultural output [30,31]. Due to global climate change, oomycetes may become more problematic as their geographic distribution shifts polewards [32].

Oomycetes are more closely related to brown algae and diatoms than true fungi, and they differ physiologically from fungi [26,33]. Even though they have superficial similarities, the structural differences between fungal and oomycete pathogens mean that many fungicides do not work against them [33]. Cellulose and β-glucans primarily compose the cell walls of oomycetes, distinguishing them from the chitin-rich cell walls of fungi [34]. Oomycete lineages, however, differ from each other in the compositions of carbohydrates, including N-acetylglucosamine (the monomer of chitin) within their cell wall [35]. For example, N-acetylglucosamine is absent in the cell wall of some oomycete lineages such as *Phytophthora*, but it composes nearly 10% in the cell wall of other oomycetes, such as those within the *Aphanomyces* genus [35]. Oomycetes are also heterogeneous in their ability to biosynthesize sterols [36,37]. A number of oomycetes, such as *P. infestans*, are sterol auxotrophs, making fungicides targeting sterol production ineffective against them [36,38].

Due to the unique challenges related to the physiology of oomycetes, it is desirable to obtain an arsenal of control strategies, including microbial biocontrol agents (MBCAs), which can provide alternatives to chemical-based plant protection products. MBCAs can work against oomycete pathogens through indirect mechanisms, such as plant priming and induced resistance, or direct mechanisms through chemical defenses, such as enzymatic, volatile organic compounds, antibiotic production, competition, and hyperparasitism, as well as combinations of the above modes [39,40,41,42,43]. Microbiome manipulations (see Box 1) through the addition of synthetic communities may offer treatment options for oomycete pathogens, potentially reducing a reliance on chemical fungicides. However, a number of barriers exist to the adoption of MBCAs for widespread commercial use, and there has been limited success of MBCAs in the field compared to experimental setups in laboratory environments [44,45]. While theory gives some information regarding circumstances under which biocontrol agents might work [46], their applicability in the field depends on our understanding of ecological dynamics in natural populations. The evaluation of MBCAs’ ecological characteristics, including their distribution, culturability, phenotypic plasticity, effectiveness, and persistence in natural conditions, can help dictate when and where they are appropriate to use [47,48,49,50]. This review aims to identify and discuss relevant ecological dynamics that may influence the effectiveness of microbiome-based treatments against pathogenic oomycetes. Additionally, we identify studies that consider the microbiome in the context of the biocontrol of oomycete plant pathogens and for which ecological processes are relevant.

## 2. Ecological Processes and Their Effects on the Introduction and Success of Microbial Biocontrol Agents

### 2.1. Community Assembly Processes and Context

Community assembly processes, which are series of events that affect species’ identity and abundance within a community, may influence the establishment and expression of MBCAs on plants. The habitats for microorganisms on the above-ground part of the plant, the phyllosphere, or below-ground part of the plant, the rhizosphere, have different characteristics, ecological constraints, and abiotic and biotic factors, such as UV, wind, rain, insect vectors, and human activities, that can affect how microbial communities establish [51,52]. Plant phyllosphere and rhizosphere community structure, including diversity, richness, and the presence of particular species, has a role in determining the community invasibility or the outcome of the introduction of additional species [53,54,55,56]. Recent work focused on utilizing the plant microbiome to combat plant diseases has largely consisted of engineering particular microbiomes through inorganic or organic amendments or direct applications of microorganisms. Soil amendments consisting of inorganic material (i.e., lime and vermiculite) or organic material (i.e., biochar, organic waste, and manure) are added to encourage the establishment of a favorable microbiome through the construction of a suitable microhabitat [57,58,59]. Such organic amendments that help recruit and/or retain disease suppressive or growth promoting microorganisms can be considered prebiotics [58,59,60]. The direct applications of derived or engineered communities to shift the microbiome can be considered more like a probiotic treatment [60,61,62,63]. The success of pre- and probiotics are driven by the ecological dynamics of the system. The contribution of both deterministic (niche driven) and stochastic (neutral) processes to community assembly [64] may help explain the success of biocontrol organisms from various sources in different situations. In this section, the following ecological concepts and their potential effect on the establishment and success of MBCAs are explored: priority effects, genotype- and environment-dependent expression, species interactions, competition, and niche use.

Box 1 **Glossary of terms used to describe ecological processes or concepts that may aid in the understanding of or affect biocontrol microorganism establishment.**

**Colonization/Establishment**: The ability of an organism to start a new population in a novel or uncolonized habitat.**Community invasibility**: The ability for a species or population to establish itself and grow in a habitat occupied by a community of different organisms.**Dispersal**: The movement of organisms among and within habitats and habitat patches.**Ecological drift**: The change of species abundances within a community over time due to stochastic processes.**Environmental filters**: The selection of a subset of species that can withstand the abiotic conditions of an environment and determines a community.**Metacommunity**: An interconnected community of multiple species that is spread across different habitat patches.**Metapopulation**: An interconnected population of one species that is spread across different habitat patches.**Neutral theory in ecology**: Simplest model possible for biogeographical patterns of diversity in which all species are first assumed to be equally capable of competing. Neutral drift and random dispersal are main factors that shape community assembly.**Niche theory**: The distribution of species due to their n-dimensional hypervolume or the space corresponding to species’ requirements (habitat, environmental conditions, food, etc.). This framework is contrary to the neutral theory and suggests certain species are better suited for particular environments and community assembly processes are deterministic.**Patch dynamics**: The interconnectedness of populations and communities across mosaic landscapes composed of heterogeneous patches or habitats. The distribution, size, and interconnectedness of patches have an effect of biodiversity maintenance.**Phenotypic plasticity**: The variation of phenotypic traits observed due to differences in environmental conditions. Variation in phenotypic plasticity gives rise to genotype by environment (G×E) interactions.**Priority effects**: The occurrence of earlier arrivals to a habitat having an advantage for establishment compared to later arrivals during community assembly.**Reaction norm**: Pattern of a genotype’s trait expression across different environments. The slope of the reaction norm gives information regarding how responsive a trait is to environmental variation.**Source-sink dynamics**: An aspect of patch dynamics where some high quality patches represent sources of species or populations while other poor-quality patches represent sinks.

#### 2.1.1. Priority Effects

Priority effects appear to influence the plant microbiome [65,66,67], so that early arrivals are better able to colonize and establish themselves on plants. Priority effects are borne out in the results of soil swap experiments, which show that the soil influences the bacteria within the phyllosphere [68]. The importance of priority effects and the early establishment of the microbiome indicate that seed or soil treatments may be a useful tool for microbiome manipulations [69,70]. The plant microbiome has been described as having a source to sink gradient from the ground up [71], meaning that soil serves as a reservoir for microbial colonization of above-ground plant parts. These source–sink dynamics of plant microbiota may also correspond to the role that soil can play in disease suppression or induction [72,73,74,75]. Priority effects can influence the composition of the community by resulting in alternative stable states or different final community outcomes, ultimately affecting function or epidemiological outcome [67,76]. By capitalizing on the influence of priority effects, microbial inputs at an early plant stage may have large effects in community assembly and on disease protection [69]. Community assembly can also be governed by multiple processes, including host genotype [77,78] and its interaction with priority effects [66]. Such assembly dynamics can, in turn, influence the community invasibility and dictate the establishment and success of the biocontrol agent [79].

#### 2.1.2. Community Dynamics, Resource Competition, and Niche Space

Once a community is established, some species may act as keystone or hub species and have an outsized effect on the microbiome. The oomycete pathogen *Albugo laibachii* has such a disproportionate influence on community assembly in infected plants because it suppresses other microbes on the plants [80]. In addition to changing the diversity of the microbial community, an infection can also alter the composition of the microbial community. An oomycete infection of *Phytophthora parasitica* in tomatoes increased the relative abundance of members of the bacterial phylum *Bacteroidetes* [81], and their growth was thought to be promoted due to increased pectin degradation caused by the infection. Aside from an infection shifting community dynamics, a loss of community members can also affect disease suppression. The loss of relatively rare bacterial species has been shown to reduce antifungal volatile organic compounds (VOCs)—secondary bacterial metabolites that can move through air or water and can inhibit the growth of pathogenic organisms, suggesting that microbial community interactions may drive volatile production and affect the biocontrol activity of the community [82]. Such interactions among community members and pathogens that modulate gene expression have implications for biocontrol success. For example, the biological control agent *Pseudomonas fluorescens* In5 increases expression of secondary metabolites in the presence of the plant fungal pathogen *Rhizoctonia solani*, but not the oomycete pathogen *Pythium aphanidermatum* [83]. Instead, *P. aphanidermatum* reduced important lipoproteins that help *P. fluorescens* In5 control pathogens [41]. Therefore, the presence of a particular microbial community member or co-infection can cause shifts in microbial communities and, in turn, may affect the establishment and persistence of or protection conferred by a biocontrol agent.

Resource competition within bacterial communities can also affect pathogen invasion success [43,84], suggesting that community architecture can have a large impact on plant–pathogen interactions. Plants hosting microbial communities with greater niche overlap showed stronger pathogen resistance, which corresponds to a decrease in susceptibility to pathogen invasions in plants harboring more diverse microbial communities [53,54,55,56]. By utilizing concepts about niche theory, exploring the niche breadth of candidate microbial agents can also contribute to a better understanding of the success of their introduction. For example, the lifestyle of the oomycete—whether it regularly colonizes the rhizosphere or phyllosphere of the plant—may influence the strategy for plant protection. A subset of phyllosphere and root microbiota is transferable between the two habitats while others are niche-specific [85], so the design of treatments should consider the target area of inoculation (i.e., seed or foliar application) and the success of colonization. An analysis of genomes from plant-associated bacteria shows that their genomes include more carbohydrate metabolism functions [86], possibly indicating that bacteria derived from plants are better suited to establish and persist on a plant than non-plant associated microorganisms.

Available niche space and the ability to outperform pre-established microbes may influence success of the introduction of biocontrol agents. Therefore, the persistence and stability of an introduced biocontrol agent should be monitored. Even if an ideal community to suppress disease is achieved, a main ecological question remains regarding how it is maintained. Within ecological communities, complex interactions arise that make it difficult to control subsequent processes. For example, intraspecific oomycete and bacterial–oomycete interactions can facilitate infections through virulence signaling or the facilitation of opportunistic infections [81,87,88]. Factors such as plant age, resident microbial community, and other environmental aspects can shape the microbiome of a plant [71,89] and may potentially affect the establishment, persistence, and influence of transplanted microbes. However, even if some introduced microorganisms do not persist, they can have lasting effects on plants. For example, after 100 days, willows inoculated with different starting rhizosphere soil showed differences in growth according to their initial microbial inoculation despite the convergence of the microbiome [90]. Therefore, the context of these intricate interactions could change the disease outcome.

#### 2.1.3. Phenotypic Plasticity

**Figure 1:**
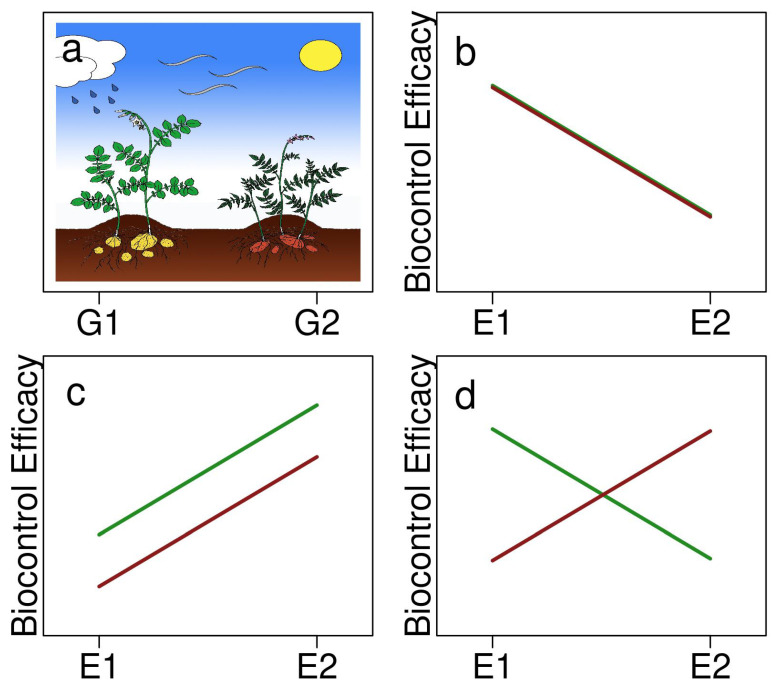
A representation of two potato genotypes, (**a**) G1 (green) and G2 (red), and (**b**–**d**) three possible outcomes of an MBCA’s biocontrol efficacy (trait phenotype) across two different environments (E1 and E2) as illustrated by reaction norms showing: (**b**) the loss of efficacy in E2 but no difference between genotypes, (**c**) reduced efficacy in E1 and G2 but parallel responses across genotypes, and (**d**) G×E interaction, where G2 is more effective in E2 and G1 more effective in E1.

Phenotypic plasticity can cause context-specific outcomes, which generally arise from the effect of genotype by environment (G×E) interactions on a trait’s expression. When a trait exhibits plasticity, its expression changes depending on environmental conditions. The microbiome can be considered an “extended genotype” due to the genetic repertoire included in the plants own genome as well as its microbiota [91], and it can also exhibit plasticity in the expression of its traits [89]. Once an MBCA is established, it is also subject to G×E interactions, which can affect its effectiveness to control plant diseases [92]. In terms of environmental effects, certain functions or characteristics of MBCAs can be turned on or off depending on various environmental factors [48,49]. Genotypic effects from the plant may influence microbiome traits involved in microbial recognition or triggering an immune response, which could in turn, affect the MBCAs’ disease suppressiveness [92]. Together, G×E effects and the phenotypic plasticity that can influence MBCAs’ efficacies make it imperative to test a variety of environmental and genotype combinations before deploying MBCA solutions. Environmental-dependent expression of biocontrol agents can give rise to non-parallel reaction norms (Figure 1), meaning that G×E interactions are apparent, and potentially, some G×E combinations are much more effective than others. Context dependency may also be due to the influence of the environment serving as the source of the plant’s microbiota [89,93]. This means that the location of the plant and correspondingly available microbes, including their genetic material that is incorporated into the microbiome, can feedback and influence an MBCA’s success.

### 2.2. The Effect of Spatial Dynamics on Microbial Biocontrol Agents: Metapopulation and Metacommunity Perspectives

Spatial dynamics can have a role in the persistence and success of microbiome manipulations as well as the occurrence of plant pathogens. Epidemiological models and studies have long accounted for spatial scales to study disease pressure in human diseases, including work on ‘landscape epidemiology’ dating back to the 1930s [94]. Landscape and spatial lens have also been used to investigate crop pests [95,96], and the advancement of drone technology will likely assist such investigations in the future [97,98,99,100]. A number of oomycete pathogens have demonstrated spatially- and density-dependent patterns of disease occurrence, including *Pythium* and *Phytophthora* species [101,102,103]. More recently, building on population spatial dynamics, ecological frameworks including metapopulation and metacommunity perspectives have been described as a useful tool through which to analyze the interaction of multiple populations or species across a landscape [104]. Such perspectives may contribute to a better understanding of the dynamics within a pathosystem and help to determine how to decrease disease pressure [105,106].

Spatial analyses of metapopulations show that increasing distance between host plant fields and using a mixture of resistant and susceptible host plants can reduce disease severity [103]. Although a metapopulation perspective can be informative to analyze plant–pathogen interactions and their evolution [107,108], it focuses on the distribution and dynamics of single species in landscapes. However, within that same scale, multiple pairs of interacting species comprise a metacommunity [104,109], which can help offer a framework to examine not only host–pathogen interactions, but also those of biocontrol organisms and multiple members of the plant microbiome.

The metacommunity framework, which incorporates interacting species at multiple ecological, spatial, and evolutionary scales [104,110], can be useful to examine plant–pathogen dynamics [111]. Within a metacommunity lens, stochastic processes such as drift and dispersal are considered, which can greatly affect pattern of biodiversity in small communities [112]. Metacommunity dynamics can also affect the evolution of pathogens due to horizontal gene transfer with fungal cohabitants, which has been documented in the following oomycetes *Phytophthora ramorum*, *Phytophthora sojae*, *Phytophthora infestans*, and *Hyaloperonospora parasitica* [113]. Spatial dynamics of metacommunities that include pathogenic organisms should not only be considered on a macro (between field and plant scales), but also on a microscale, including within the rhizosphere [114] or plant.

**Figure 2:**
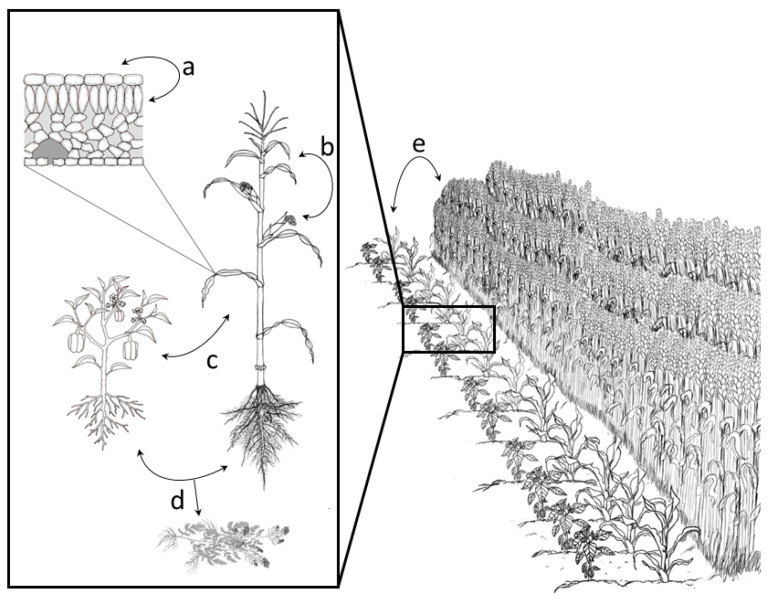
Illustration demonstrating interacting spatial scales within agricultural systems that can be considered through a metacommunity framework. Exchange of microorganisms among these spatial scales can impact the success of microbial biological controls. These spatial scales include dispersal of microorganisms (**a**) within leaf microhabitats, including within the leaf and on the leaf surface; (**b**) between leaves on the same plant; (**c**) between neighboring plant hosts of different species, shown here within an intercropping system; (**d**) from pre-cropping material (either due to legacy effects in the soil or due to remaining plant debris); and (**e**) from neighboring fields.

A metacommunity perspective can help inform biocontrol applications as the dynamics of an MBCA’s population is likely to be influenced by multiple interactions among other members of the local and regional pool of microorganisms (Figure 2). Recent work has described the utility of using metacommunity theory to study symbiont evolutionary ecology [115], and such interactions among symbionts, such as the timing and sequence of multiple infections, can influence parasitic epidemics within plants [67]. In the case of agricultural settings, the regional species pool is influenced by the previous crop, which in turn, affects oomycete and bacterial communities [116,117].

Intercropping can be one way to expand the regional species pool and increase patch heterogeneity, which can help to decrease disease pressure through additional species’ interactions. The prevention of infection from potential pathogens may depend on having a more diverse metacommunity from which mutualistic microbial members can arise [118]. Spatial crop configurations that produce more diverse microbial communities [119,120] may help disease suppression through potential niche overlap between endophytes and pathogens [121]. Additionally, intercropping may contribute to reduced disease incidence through induced resistance from other plants or the production of allelopathic compounds [122]. *Phytophthora infestans* can be reduced with intercrops of cereal or grass-clover [123], and maize-pepper systems show reduced *Phytophthora capsici* severity through antimicrobial compounds secreted by maize [124].

## 3. Challenges and Limitations of Microbial Manipulations in Agricultural Systems

In the last decade, more research papers consider the microbiome in the control of oomycete pathogens (Table 1). Many of the aforementioned ecological concepts, including G×E interactions, community context, and spatial dynamics have been discussed in recent literature. These research avenues can help broaden the understanding of when MBCAs can work and in which context, but there are still many challenges with implementing effective MBCAs against oomycete pathogens.

For example, microbial communities are attributed to suppress diseases (i.e., within disease-suppressive soils), however, they are difficult to recreate in the field due to the involvement of multiple species [74]. The vast array of microbial diversity may make it challenging to create persistent alterations to a plant’s microbiome in the field. Weinhold and colleagues [151] genetically modified the wild tobacco plant (*Nicotiana attenuate*) to constitutively express antimicrobial peptide 1 (Mc-AMP1) of the common ice plant (*Mesembryanthemum crystallinum*), which selects against Gram-positive bacteria. Despite differences in microbial communities in greenhouse experiments—mainly through a reduction in beneficial bacterial, field trials showed no major alterations between microbial communities in engineered plants and normal plants. Rather than seeing systematic differences in modified plants in the field, it was found that root associated bacterial communities were altered only in subtle ways, which the authors attributed to the resilience of diverse microbial communities in nature. Such studies may help explain why MBCAs that look promising in more controlled environments, such as greenhouses and labs, fail in the field or yield inconsistent results [152]. These disparate effects may also be due to differences in plant physiology when grown in controlled environments compared to the field [153].

Because genotype and environment have an influence on the leaf microbiome [89], it is possible that colonization and persistence of MCBAs may vary based on these attributes and their interactions. Therefore, it would be unlikely to find a ‘silver bullet’ that would provide protection against oomycete pathogens under varying host genetic background and ambient environmental conditions. Due to these constraints, the application of MBCAs for a disease can benefit from a systems approach [79] to try to better select optimum conditions and genotype combinations for the use of an MBCA.

Evolution of pathogen strains and adaptation to biocontrol agents is another factor that may change dynamics of host–pathogen interactions [154], and many questions remain about evolution of resistance to biocontrol agents. Multiple infections of *Albugo candida* may have enabled host range expansion through genomic introgressions [88]. In this case, the primary infection reduces the defense mechanisms of the host, allowing for a secondary infection of *A. candida* and genetic exchange and the production of new and more challenging pathogens. Horizontal gene transfer (HGT) among oomycete and fungi may have enabled oomycetes to become successful plant pathogens [113], and HGT among co-occurring fungi and oomycetes may serve as an additional genetic reservoirs to provide resistance to biocontrol agents. Another frontier of the microbiome is its viral constituents. Oomycete plant pathogens have been found to harbor viruses [155] that, in some cases, may also amplify their virulence by increasing sporulation [156] and possibly interfering with host–pathogen interactions.

## 4. Outlook

Microbiome manipulations can help plant hosts resist diseases [157,158] and may provide a powerful tool to help protect plants from oomycete pathogens. In co-evolving, natural systems, the microbiome may provide additional genetic tools to help its host adapt to disease. A challenge in agricultural systems is that they are not representative of natural host–pathogen systems because of the alteration of co-evolutionary dynamics. Typically, arms races can ensue in natural systems with sexually reproducing hosts, whereby the host acquires adaptations to enable it to contend with a particular pathogen and subsequently the pathogen adapts to overcome these new arms [159]. In natural systems, the microbiome may contribute to the genetic arsenal to combat oomycete pathogens. In agricultural systems, cultivars of the host plant are artificially selected for particular characteristics usually related to commercial desirability. As every generation is reset with artificially selected cultivars, the evolutionary capabilities of host–microbial partnerships may be limited due to reduced opportunities for vertical transmission and selection for pathogen suppressive traits. More work is incorporating the microbiome in breeding research including for disease resistance [6,125,160].

Recent studies have accounted for the phylogenetic distance of host plants on yield outcomes and soil microbiome community [161], and although effects on yield have not been shown, changes in soil microbiome community are apparent. Such studies help unite concepts of evolutionary ecology and microbial ecology to better understand how microbes can be utilized in agricultural practices against oomycete pathogens. Selection on soil microbiomes was shown to influence flowering traits [162], suggesting that the engineering of the microbiome for disease resistance may also be possible.

Applications of microbial consortia rather than just one species could help lead to resilient communities that have disease-fighting capacity—either through induced resistance or through direct antagonism [40,163]. In a host-mutualist context, pathogenic microorganisms can otherwise be kept in check by interactions with other members of the microbiome [118]. Although it has been argued that most combined use of biocontrol agents results in antagonistic relationships between MBCAs and only one of the MCBAs is responsible for most of the suppression [164], a number of microbial consortia have shown improved results against oomycete pathogens [42,55,165]. These conflicting results suggest that a community approach or the application of multiple microbes could provide more protection rather than a single strain in certain circumstances. Although the dynamics of a community of MBCAs, rather than just a few strains or species, would be more intricate to study, an additional obstacle is the registration procedure of MBCAs. Registration of single strains is already challenging, and due to the time and cost associated in registering MBCAs, most are often on the market as biofertilizers [166]. Therefore, the adaptation of registration procedures to also account for communities would be needed before such control strategies could be implemented.

The success of biocontrol solutions is more likely to occur if an MBCA persists after its application, so identifying factors or assembling communities that facilitate persistence is an important goal. According to Kinnunen and colleagues [167], a successful establishment of a new microbial community member is defined as the maintenance of a metabolically active population for a significant period of time, and it can depend on niche space, stochastic processes such as drift, and community diversity. Including members that aid in the formation of biofilms—structured microbial communities embedded in an extracellular matrix—may facilitate the biocontrol of oomycete pathogens [168,169,170]. Biofilms are thought to contribute to the control of pathogens through diverse means such as provoking induced systemic resistance, producing antimicrobial compounds, and excluding pathogens from niche space [171]. However, biofilm formation can also assist *Phytophthora parasitica* infections [172], and their assistance or inhibition of infection may be situational and depend on the characteristics of the biofilm and its inhabitants. Additional work is needed to help elucidate and differentiate biofilm characteristics that aid in protection and in infection. Aside from the impact of biofilm formation, the identification of other biotic and abiotic factors to enable the success of MBCAs in different environment could help direct the successful establishment of an MBCA. Due to environmental effects, more focus on functional rather than taxonomic attributes of the microbiota is needed [173].

Biocontrol solutions need individualized solutions for plant genotype, local environment, and local microbial constituents. Such customized methods for pathogen control take more work to understand effective treatments under different situations, and an “one size fits all” model for biocontrol solutions is not realistic. Additionally, given the ability for pathogenic organisms to adapt, solutions must be pliable. Although there are a number of obstacles to implementing microbial biocontrol solutions on a larger scale, the societal desire to decrease pesticide use can help derive the necessary activation energy to find microbiome-based biocontrol solutions.

## Figures and Tables

**Table 1:** Primary research papers with the search terms ‘microbiome’, ‘oomycete’, and ‘control’ were assessed to determine main research areas focusing on microbiome for biological control. The articles were categorized according to their incorporation of ecological concepts or discussion of control mechanisms in relationship to the microbiome. Search was conducted using Elsevier’s Scopus database and Web of Science and includes literature from 2013 to 2020 (see Supplementary Materials for more information).

Ecological Concept and/or Mechanism of Control	Disease or Disease Taxon	Crop ${}^{a}$	Experimental Context	Main Experimental Focus	Ref.
Influence of genotype × environment interactions	Root rot (*Aphanomyces* & *Pythium* spp.)	Pea (*Pisum sativum* L.)	*In planta* (growth chamber), on-farm	Screened resistance of pea genotypes in sterile or infected soil	[125]
		Lentil (*Lens* spp.)	*In vitro*, *In planta* (growth chamber)	Assessed commercially available products containing *Trichoderma* spp. on root colonization and plant growth promotion	[77]
	Seedling rot (*Pythium ultimum* var. *ultimum*)	Soybean (*Glycine max* L. Merr)	Field experiments	Conducted trials with four soybean genotypes across different treatment regimes in high and low disease pressure sites	[126]
	Apple replant disease (*Phytophthora* & *Pythium* spp.)	Apple (*Malus domestica*)	*In planta* (greenhouse)	Assessed different *Brassica* seed meals and rootstocks	[127]
Genotype effect on induced resistance	Downy mildew of grapevine (*Plasmopara viticola*)	Grapevine (*Vitis vinifera* L.)	*In planta* (greenhouse)	Tested *Pseudomonas fluorescens* strain in two cultivars	[128]
Influence of environmental conditions on the microbiome and/or pathogen control measures	Foliar and crown rot (*Phytophthora capsici*)	Summer squash (*Cucurbita pepo* var. *cylindrica* L.)	*In planta* (greenhouse)	Evaluated different composts, including one *Trichoderma*-enriched compost	[129]
	Apple replant disease (*Phytophthora* & *Pythium* spp.)	Apple (*Malus domestica*)	Sampling from orchards	Evaluated the effect of soil physical properties with seed meal amendments	[130]
	Various soil-borne plant oomycete pathogens	Potato (*Solanum tuberosum* L.)	Field experiments	Evaluated the effect of soil fertilization on fungal and oomycete pathogen- and mycorrhizal communities	[131]
	*Aphanomycetes* & *Pythium* spp.	Rice (*Oryza sativa* L.)	*In planta* (microcosms in 50 mL centrifuge tubes)	Assessed effects of different biochar soil amendments on relative abundance of oomycetes	[132]
Induced resistance	*Pythium ultimum*	Romaine lettuce (*Lactuca sativa* L. var. *longifolia*)	*In planta* (hydroponic systems)	Evaluated treatments with *Pseudomonas chlororaphis*, UV irradiation and different media	[133]
Interaction of pathogen and biocontrol treatments	Root rot and damping-off (*Pythium* spp.)	*Brassica* microgreens: Arugula (*Eruca sativa* Mill.), kale (*Brassica oleracea* var. *sabellica* L.), radish (*Raphanus raphanistrum* subsp. *sativus* L.), and mustard (*Brassica juncea* L. Czern) microgreens	*In planta* (hydroponic and tray experiments)	Tested commercial products: Companion^®^ (*Bacillus subtilis* GB03), Triathlon BA^®^ (*Bacillus amyloliquefaciens* D747), and RootShield Plus^®^ (*Trichoderma harzianum* KRL-AG2 and *Trichoderma virens* G-41)	[134]
Direct antagonism from members of microbiome	Potato late blight (*Phytophthora infestans*)	Potato (*Solanum tuberosum* L.)	*In vitro*, *In planta* (leaf disks)	Tested VOCs	[135]
		Potato (*Solanum tuberosum* L.)	*In planta* (leaf disks)	Tested effect of sulfur-containing VOCs	[136]
		Grapevine (*Vitis vinifera* L.)	*In vitro*	Screened microbial isolates for biocontrol properties	[137]
	Foot rot (*Phytophthora capsici*) & *Pythium myriotylum*	Black pepper (*Piper nigrum* L.)	*In vitro*, *In planta* (shoot cuttings)	Used chemically synthesized volatiles from a *Pseudomonas putida* strain	[138]
	Root rot (*Pythium aphanidermatum* & *Phytophthora capsici*)	Diverse cucurbits	*In vitro*	Screened microbial isolates from seed endophytes	[139]
	Crown rot (*Phytophthora capsici*)	Zucchini (*Cucurbita pepo* L.)	Field experiments	Tested commercially available and experimental biocontrol agents and composts	[140]
	Root rot (*Phytophthora cinnamomi* & *Phytophthora nicotianae*)	Lavender (*Lavandula angustifolia* var. Hidcote), Olive (*Olea europaea* L)	*In vitro*, *In planta* (greenhouse trials)	Screened *Trichoderma* species isolated from rhizospheres (also for induced resistance)	[141]
	Root and/or crown rot (*Phytophthora cinnamomi*)	Avocado (*Persea americana* Mill.)	*In vitro*	Screened microbial isolates	[142]
		Strawberry (*Fragaria* × *ananassa*) plants	*In vitro*	Tested volitales from an *Arthrobacter agilis* strain	[143]
		Avocado (*Persea americana* Mill.)	*In vitro*	Screening of microbial isolates from rhizosphere	[144]
	*Phytophthora* & *Pythium* spp., *Phytopythium vexans*	Olive (*Olea europaea* L)	*In vitro*	Screened microbial isolates from root endophytes	[145]
	Damping off/root rot (*Pythium aphanidermatum*)	Cucumber (*Cucumis sativus*)	*In vitro*, *In planta* (seedlings)	Tested recruited microbiome from vermicomposted dairy manure	[72]
	Downy mildew (*Sclerospora graminicola*)	Pearl Millet (*Cenchrus americanus* L.)	*In planta* (germination tests and greenhouse trials)	Screened microbial isolates	[146]
Alternative hosts/reservoirs of pathogens (metapopulation dynamics)	Potato late blight (*Phytophthora infestans*), root rot (*Pythium* spp.)	Potato (*Solanum tuberosum* L.)	*In planta* (detached leaf, root infection assays)	Collected oomycete communities from wild *Solanum* species	[147]
	*Pythium* & *Phytophthora* spp.	Not applicable	Sampling from semi-natural and natural ecosystems	Sampled diversity and distribution of oomycetes across landscapes	[148]
Spatial structure and/or microbial community dynamics	*Pythium volutum*, *Pythium* sp. F86, and *Lagena radicicola*	Corn (*Zea mays* L.)	Field experiments, *In planta* (pathogenicity assays with isolates of *P. volutum*)	Evaluated the effect of rye cover crop termination on fungal and oomycete communities	[149]
	Root rot (*Phytophthora cinnamomi*)	Cranberries (*Vaccinium macrocarpon* Ait.)	*In vitro*	Screened microbial isolates for VOCs in monocultures and bacterial-fungal co-cultures	[150]

^a^ Source of biocontrol organisms or crop in which biocontrol was tested.

## Supplementary Materials

The following are available online at https://www.mdpi.com/article/10.3390/plants10122697/s1.
