# Supplementary material for: Ecological Dynamics and Microbial Treatments against Oomycete Plant Pathogens
Source: Plants (Basel). 2021 Dec 8;10(12):2697. doi: 10.3390/plants10122697 (PMC8707103; doi:10.3390/plants10122697)
Supplement: Supplementary file 1 [file plants-10-02697-s001.zip › plants-1384034-supplementary.pdf]

Supplementary Material: Ecological dynamics and their effect on microbial treatments against oomycete plant pathogens

A review of papers using the search terms "microbiome, oomycete, and control" limited to agricultural subject area was conducted on December 2, 2020 using Elsevier's Scopus database and Web of Science. The purpose of the literature search was to determine main research areas that consider the microbiome in the context agricultural oomycete disease control. The relevant papers were summarized based on control measures applied or evaluated and/or incorporation of ecological concepts into the studies.

The search resulted in 157 article hits, of which duplicates and non-relevant literature were removed. Articles were determined to be non-relevant if the articles were not original research or not focusing on agricultural research. Additionally, papers were removed that only focused on fungal pathogens rather than oomycete pathogens. After this selection process, 28 articles satisfied these criteria. These articles were then analyzed based on the discussion of ecological concepts and/or primary mode of action of biological controls (i.e. direct antagonism, induced resistance).
